# Environmental scan and evaluation of best practices for online systematic review resources

**DOI:** 10.5195/jmla.2018.241

**Published:** 2018-04-01

**Authors:** Robin M. N. Parker, Leah M. Boulos, Sarah Visintini, Krista Ritchie, Jill Hayden

**Affiliations:** Evidence Synthesis and Information Services Librarian, W.K. Kellogg Health Sciences Library, and Department of Community Health and Epidemiology, Dalhousie University, Halifax, NS, Canada; Evidence Synthesis Coordinator, Maritime SPOR SUPPORT Unit, Halifax, NS, Canada; Evidence Synthesis Coordinator, Maritime SPOR SUPPORT Unit, Halifax, NS, Canada; Assistant Professor, Faculty of Education, Mount Saint Vincent University, Halifax, NS, Canada; Associate Professor, Department of Community Health and Epidemiology, Dalhousie University, Halifax, NS, Canada

## Abstract

**Objective:**

Online training for systematic review methodology is an attractive option due to flexibility and limited availability of in-person instruction. Librarians often direct new reviewers to these online resources, so they should be knowledgeable about the variety of available resources. The objective for this project was to conduct an environmental scan of online systematic review training resources and evaluate those identified resources.

**Methods:**

The authors systematically searched for electronic learning resources pertaining to systematic review methods. After screening for inclusion, we collected data about characteristics of training resources and assigned scores in the domains of (1) content, (2) design, (3) interactivity, and (4) usability by applying a previously published evaluation rubric for online instruction modules. We described the characteristics and scores for each training resource and compared performance across the domains.

**Results:**

Twenty training resources were evaluated. Average overall score of online instructional resources was 61%. Online courses (n=7) averaged 73%, web modules (n=5) 64%, and videos (n=8) 48%. The top 5 highest scoring resources were in course or web module format, featured high interactivity, and required a longer (>5hrs) time commitment from users.

**Conclusion:**

This study revealed that resources include appropriate content but are less likely to adhere to principles of online training design and interactivity. Awareness of these resources will allow librarians to make informed recommendations for training based on patrons’ needs. Future online systematic review training resources should use established best practices for e-learning to provide high-quality resources, regardless of format or user time commitment.

## INTRODUCTION

Systematic reviews are a cornerstone of evidence-informed decision making that has dominated health professional practice and education for more than twenty years. There are several reasons researchers and trainees conduct systematic reviews: reviews serve to familiarize learners with new areas of research; they may resolve conflicting evidence; they do not require an extensive research budget for equipment or material; and there is usually no need for research ethics approval. In addition, the generally high citation rate of systematic reviews makes them an appealing publication type for researchers and journals. In academic and clinical settings, health librarians can support systematic review projects by developing and executing the comprehensive literature search [1–4] and educating the review team regarding methods. In addition to providing in-person instruction, librarians frequently direct reviewers to training resources and guidance documents, including web-based learning tools.

The effectiveness of e-learning in health care contexts has been examined extensively in various settings for diverse learners, including recent reviews [5–13]. However, it is unclear if the evidence on online learning has been used to guide available delivery of systematic review training in the web-based environment. There is research examining the performance and usability of online instruction for searching and information literacy [14–16] and evaluating online modules for evidence-based practice (EBP) [17]. For example, the Foster et al. environmental scan used a standard rubric and multiple levels of assessment to evaluate Internet-based instruction in EBP [17]. To date, however, a similar evaluation of online instruction for systematic review methods has not been published.

Although there are brief mentions in the literature of online training resources to learn systematic review methods [16, 18–21], there have been no attempts to comprehensively evaluate the available resources against best practices. The objective of this study was to conduct an environmental scan and assessment of online systematic review training resources in order to describe available resources and to evaluate whether they follow current best practices for online instruction.

## METHODS

The authors conducted an environmental scan for online systematic review educational resources using several strategies. Our methods to identify potential online training resources combined approaches used by others, such as exhaustive YouTube searches [22], screening of Google searches to the point of exhausting the relevant results [22], and targeted searching of websites [23].

The broad Google search consisted of:

((“systematic review” OR “scoping review” OR “evidence review” OR “knowledge synthesis” OR “evidence synthesis”) online teaching OR course OR workshop OR seminar OR education OR training OR module).

Following the approach described by Galipeau and Moher [24], we scanned the first twenty results for relevance and made selections for further investigation based on the eligibility criteria. If the first twenty results had a result to be included, then we reviewed the next twenty until a page was reached with no eligible results [24].

We also searched websites of organizations recognized for producing evidence syntheses or providing health research methods training by using a targeted Google search string with variant terms for systematic reviews and training. These targeted searches included Cochrane entity websites, North American medical school websites, massive online open course (MOOC) platforms, and knowledge synthesis organizations, such as the Agency for Healthcare Research and Quality and the National Collaborating Centre for Methods and Tools (supplemental Appendix A). Searches were completed during the summer of 2015.

### Selection criteria

Selection criteria focused on training resources with specific content, format, availability, and language characteristics.

- Content: We included training resources if their content pertained to at least three of six systematic review steps: (1) defining a research question and/or creating a protocol, (2) conducting a rigorous search, (3) determining selection criteria, (4) performing a critical appraisal and/or risk of bias assessment, (5) extracting data, and (6) performing analysis and/or creating an in-depth report.
- Format: Inclusion criteria specified online courses, videos, and web tutorials or modules. Online courses were defined as synchronous or asynchronous with the inclusion of mediation by an instructor, whereas web modules were defined as stand-alone resources that could be navigated by learners at their own pace. Resources were excluded if they consisted of face-to-face or blended learning formats or noninteractive, text-based information (e.g., journal articles or books).
- Availability: Resources that were available (for free or for a fee) to the public or to a group with open membership were included. Resources were excluded if they were restricted to specific employees or students of an institution or if we were unable to gather necessary information on the content or delivery of the resource through searching publicly available material or by contacting the creators.
- Language: Only resources provided in English were included.

We reviewed resources in duplicate and resolved conflicts by consensus as a group. When we were unsure about inclusion, resources were tentatively included until more information regarding content or delivery could be acquired from the creators. One author contacted creators via email and sent a follow-up email within six weeks if no response was received upon initial contact. If no response was received after both emails, resources were excluded due to insufficient information.

### Data extraction

We extracted the following information from the included resources: name of the resource, institutional host or creator, uniform resource locator (URL), access (e.g., available on demand or via scheduled offering), disclosed intended audience, type of host (e.g., university or government department), country of origin, format, duration, and cost. Data were extracted by one team member and checked by a second team member.

### Evaluation

Each of the included resources was reviewed independently in duplicate, based on the predefined point system for quality in four domains developed by Foster et al. and based on the Quality Assessment of Digital Education Material (QuADEM) approach: “Content,” “Design,” “Interactivity,” and “Usability” [17, 25]. Responses were exported into Microsoft Excel, inter-rater agreement was calculated, and conflicts were resolved by a third team member. Three questions were “select all that apply” with 1 point awarded for each selection, and the remaining 23 questions could be answered with “yes” (1 point), “no,” or “can’t tell from available info” (0 points), or in most questions, “somewhat” (0.5 points). The total score for the rubric (37 points) deviated slightly from Foster et al.’s model (38 points) due to an alteration of the total possible points in question 7 regarding the types of interaction [17]. Where Foster et al. assigned the question 6 points (5 points for various activities and 1 point for “other”) [17], we limited this score to 5, with the possible point for “other” serving as an alternate, rather than additional, tally (supplemental Appendix B).

1. To evaluate Content, we indicated which of the six steps of the systematic review process (as determined for the selection criteria) each resource covered. As with the reference rubric, the actual topics covered were not given a scoring weight but were extracted for analysis of topic coverage [17]. Evaluation criteria pertained to credibility, relevance, currency, organization, ease of understanding, focus, and appropriateness of language, and we assigned points out of 7 in this domain.
2. Design: We assigned points out of 12 for Design based on the levels of cognitive process that were encouraged by each resource, as defined by the revised Bloom’s Taxonomy, 2001 version [26]; the inclusion of learning objectives as well as their measurability and coverage; and the types of learning styles incorporated (visual, audio, or spatial).
3. For Interactivity, we assigned points out of 10, based on the level of interactivity, variety of tasks employed (e.g., clicking, performing a search, scrolling, answering quizzes, or typing in answers), appropriateness and difficulty level of tasks, and opportunities for reflection and feedback.
4. For Usability of resources, we assigned points out of 8, based on the layout and ease of navigation (e.g., use of navigation menus) and compliance with aspects of the Americans with Disabilities Act, as described by Foster et al. [17]. This meant low use of red or green, the inclusion of captions, consistent navigation, audio that could be turned off, and the ability to pause.

### Data collection

We piloted a data collection form in duplicate on three resources, which revealed inconsistency in interpretation of the evaluation questions between the reviewers. To clarify evaluation criteria, we supplemented the form with descriptions from the QuADEM Manual [25], which Foster et al. drew on when they created their original evaluation form [17]. Evaluators were also given the opportunity to leave notes in each section of the form to explain decisions or highlight sources of ambiguity in the evaluation process.

### Analysis

We described characteristics of the identified resources and the quality scores, both overall and for each evaluation domain. We compared resources and tested for statistically significant differences by conducting repeated measures analysis of variance (ANOVA) of scores for each of the four rubric categories.

## RESULTS

### Environmental scan results

**Figure 1:**
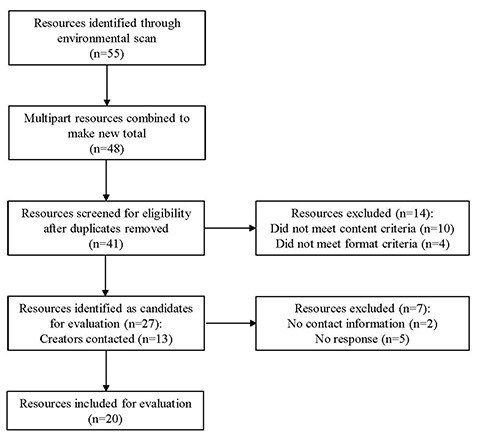
Environmental scan inclusion flowchart

Our environmental scan identified 55 resources. In cases where a resource was a single part of a multipart series or set, we combined parts to unify the set. This resulted in a list of 48 resources. We identified and removed 7 duplicates at the outset. Using the criteria outlined above, 14 resources were excluded for covering fewer than 3 of the 6 steps of a systematic review (n=10) or for being in a format that was inappropriate for this study (n=4). Of the resources that were not freely available to the public (n=15), 13 had contact information for the creators, from whom we solicited additional information. Eight responded and provided sufficient example material to evaluate the resources, while 5 resources were removed from consideration due to lack of response. Therefore, we evaluated a total of 20 online systematic review training resources (Figure 1). The full list of evaluated sources is available in supplemental Appendix C.

Researchers, health care professionals, and students were among the target audiences described, although nearly half of the resources were missing this information, and some resources were directed at more than one group (Table 1). Almost half of the included resources were produced by universities, while the rest were created by various research organizations and government agencies, including organizations specializing in knowledge synthesis and knowledge translation, professional associations, a “Centre of Excellence,” and a continuing education provider. The creator organizations were located in various countries in Europe, the United States, Canada, and Australia, reflecting the English language inclusion criteria. The formats included videos, websites or web modules, and online courses. Resources required a wide range of time to complete, from less than 1 hour to more than 200 hours over 15 weeks of sessions. Of the 20 evaluated resources, 14 were available free of charge at the time of review. Prices for the other 6 resources ranged from less than $15 USD to over $3,000 USD.

**Table 1:** Characteristics of the 20 systematic review online resources identified in an environmental scan

	n	%
Intended audience*		
Not disclosed	9	45%
Researchers	6	30%
Health care professionals	5	25%
Students	4	20%
Country		
United States	9	45%
United Kingdom	6	30%
Canada	2	10%
Australia	2	10%
Norway	1	5%
Format		
Video	8	40%
Website/web module	5	25%
Online course	7	35%
Duration		
Less than 1 hour	4	20%
1–5 hours	4	20%
5–10 hours	2	10%
10+ hours	9	45%
Variable†	1	5%
Cost		
Freely available	14	70%
Fee-based	6	30%

* The intended audience of these resources does not add up to 20 because some resources disclosed multiple audience types.

† Self-directed web module with linked resources that could take a little as 10 minutes or as many as 10+ hours.

### Evaluation results

#### Distribution of online resource quality scores

Out of a possible 37 total points, scores ranged from 13 to 34 (35% to 92%), with an average score of 22.4 (61%) and a median score of 21 (57%). Inter-rater agreement for all evaluations was 82%. There was a 1.5 average mean difference, with a standard deviation of 5.2 for total scores based on each rater’s scoring (*t*(18)=1.3, *p*=0.217), indicating high agreement between independent raters.

**Figure 2:**
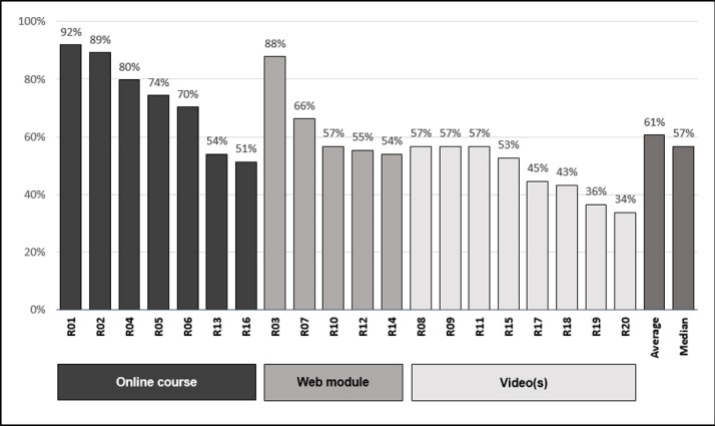
Overall scores (%) arranged by resource format

The overall scores of all 20 resources, arranged by format, are shown in Figure 2. The number assigned to each resource corresponds to its rank by overall score, where the first resource (R01) has the highest overall score and R20 has the lowest. Exact scores for each question across all 20 resources can be found in supplemental Appendix D.

#### Results by evaluation criteria

**Figure 3:**
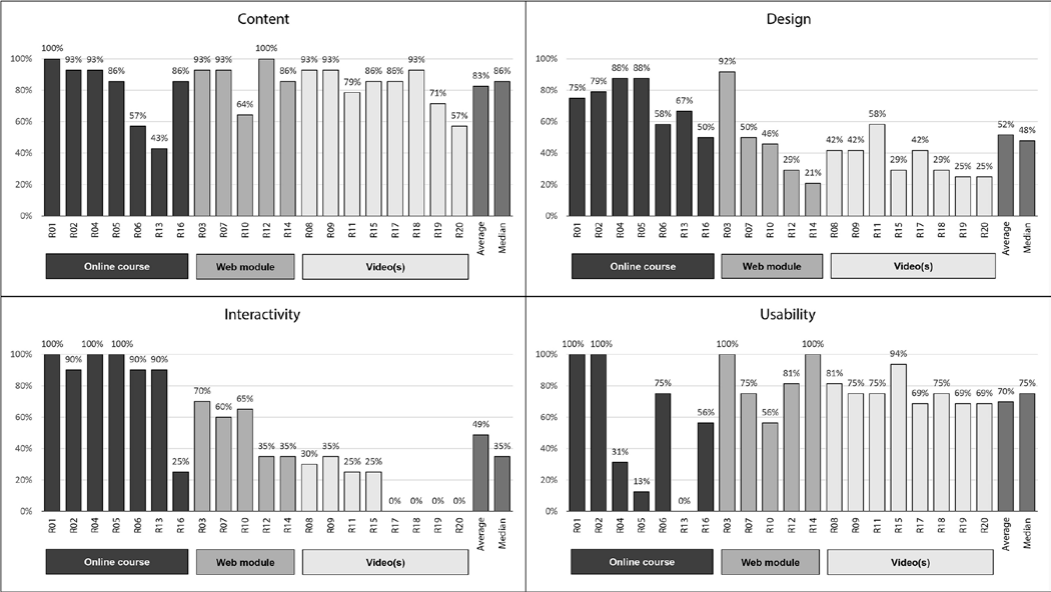
Content, design, interactivity, and usability scores (%) arranged by resource format

1. Regarding the Content domain, none of the 6 steps of a review was perfectly covered by all resources, but some version of the step classified as “Presenting findings/analysis” was covered by 19 of the resources (95%). The steps teaching how to define the research questions, conduct a thorough search, and perform an assessment of bias were all covered by 18 of the resources (90%). Twelve resources (60%) covered material on all 6 steps of a systematic review. Of the remaining 8 resources, 5 (25%) covered 5 steps and 3 (15%) covered only 3 steps. The steps that were most frequently left out were determining and/or applying selection criteria (covered by 80% of the resources) and methods or techniques for conducting the data extraction (covered by 85% of the resources). In general, although the resources had to cover at least 3 of the steps of a review to be included, the particular steps covered varied considerably across resources.
  Numerical scores were assigned for the remainder of the Content domain elements. Most, if not all, of the 20 training tools at least somewhat met criteria for credibility (n=20), relevance (n=19), currency (n=20), organization (n=18), ease of understanding (n=17), focus (n=17), and appropriate language (n=18). Scores were most mixed when we evaluated appropriateness for intended audience. The mean total Content score across all resources was 83%, with a median score of 86% (SD=15.8; range 52.8–100.0). Two resources (R01 and R12) scored 100% in this domain, and an additional 7 resources scored 93% (6.5 out of a possible 7 points) (Figure 3).
2. While Content scores were generally high, Design scores were much lower (Figure 3). Design scores reflected the resources’ coverage of Bloom’s Taxonomy levels [26], clearly explained purpose, had measurable objectives, covered those objectives, and incorporated different learning styles. Design was consistently the lowest scored domain, with no resource scoring 100%. The average score was 52%, with a median score of 48% (SD=22.9; range 20.8–91.7); over half of resources (n=12) scored 50% or lower. Only half of the 20 resources provided clearly articulated and measurable learning objectives, and even fewer (n=7, 35%) managed to completely cover those objectives in the scope of the training provided. The most common source of low scores came from our evaluation of the resources’ promotion of various levels of engagement with information, as described by Bloom’s Taxonomy [26], in which the majority of resources (n=14) covered 3 or fewer of the 6 possible levels.
3. The Interactivity domain showed the widest range of scores, with 3 resources (R01, R04, R05) scoring 100% and 4 resources (R17–R20) scoring 0 (Figure 3). This variation resulted in an average score of 49% and a median score of 35% (SD=36.9; range 0–100). Of the resources that provided opportunities for interactivity (n=16), such as clicking, scrolling, searching, answering surveys, and discussing the topic, the interactivity was generally relevant or somewhat relevant (13/15) and with an appropriate level of difficulty (yes or somewhat: 12/15) but rarely provided an opportunity to reflect on learning (6/15). Videos scored most poorly in the Interactivity domain, as they often involved little to no interactivity.
4. Usability scores resulted in the second-highest domain average at 70%, with a median of 75% (SD=27.7; range 0–100). This domain assessed the resources’ layout, ease of navigation, ability to determine learning path, and compliance with American Disabilities Act requirements regarding color, audio, captions, navigation, and pausing capabilities. The layout of resources (including visual appeal and ease of navigation) resulted in the most varied scores. Four resources (R01, R02, R03, and R14) scored 100% in this domain (Figure 3).

### Statistical analysis

Repeated measures ANOVA revealed significant differences among resources across the 4 domains (F(3, 17)=14.1, *p*<0.001). Pairwise comparisons showed that, overall, resources scored significantly higher in the Content domain than in the Design (mean difference=30.8, standard error=5.9, *p*<0.001), Usability (mean difference=12.8, standard error=5.4, *p*<0.05), and Interactivity (mean difference=33.7, standard error=9.1, *p*<0.001) domains, whereas there were no overall differences between the Design, Usability, and Interactivity domains (*p*>0.05).

Regarding other characteristics of the resources, there was a significant positive correlation (Spearman’s rho=0.47, *p*<0.05) between the time needed to complete the resource and its overall quality score. Regarding the cost of the resource, the highest overall scores occurred in the $1,000–$1,600 USD range. Although free resources tended to score lower (n=14, mean=56.08, SD=16.23) than fee-based resources (n=6, mean=71.17, SD=14.24), this difference did not reach statistical significance (*t*(18)=1.96, *p*=0.06).

### Performance of top five resources

Finally, we took a closer look at the individual domain scores for the 5 resources that received the highest overall scores (Table 2). In most cases, overall scores were affected by a relatively low score in at least 1 domain. For example, the top resource (R01) achieved a near-perfect score but did not receive full points in the Design domain. Despite achieving relatively high overall scores, the fourth- and fifth-ranking resources (R04 and R05) received 2 of the lowest Usability scores (31% and 13%, respectively) out of all resources.

**Table 2:** Characteristics of highest-scoring resources

Resource number (overall score)	Resource name	Creator	Format	Duration	Cost (USD)	Evaluation scores			

						Content	Design	Inter-activity	Usability
R01 (94%)	Comprehensive Systematic Review Training Program (Csystematic TP)*	Joanna Briggs Institute	Online course	8–40 hours	$255–$1,197	100%	75%	100%	100%
R02 (91%)	Introduction to Systematic Review and Meta-Analysis	Johns Hopkins University (through Coursera)	Online course	32 hours	Free	93%	79%	90%	100%
R03 (84%)	Online Learning Modules for Cochrane Authors*	Cochrane Training	Web module	6 hours	Free for Cochrane authors	93%	92%	70%	100%
R04 (78%)	Introduction to Systematic Review and Meta-Analysis Course	Dalla Lana School of Public Health, University of Toronto/ Knowledge Translation Program, St. Michael’s Hospital	Online course	75–110 hours	$1,195	93%	88%	100%	31%
R05 (72%)	Systematic Reviews: Diversity, Design and Debate†	EPPI-Centre	Online course	210 hours	$1,395–$3,060	86%	88%	100%	13%

* Name of resource has changed since evaluation was completed.

† No longer available as evaluated.

## DISCUSSION

Our findings revealed that systematic review e-learning resources generally performed better in the Content domain than in the Design, Interactivity, and Usability domains. Overall, performance in these domains appeared to be related to the format of the online training resources. That is, out of the five highest scoring resources, which were well designed with clear learning objectives and high levels of interactivity, four were online courses that reflected an extensive amount of time spent in their creation and effort to follow best practices in online instruction. Although the structure of online courses made it easier to receive higher scores in our evaluation rubric, it should be noted that some high-scoring videos overcame their inherent lack of interactivity and poorly defined learning objectives by having high-quality content and favorable usability characteristics, such as ease of navigation.

Most (60%) resources that we evaluated covered all steps of the systematic review process, whereas the others provided instruction only on a subset of steps. This reflected the diverse foci of the resource creators, who had different goals and intended audiences. However, it was difficult to gauge the appropriateness of the language for the 9 resources for which the audience was not well defined.

Coverage of the levels of Bloom’s Taxonomy [26] was another characteristic that had a large impact on total design score and a subsequent high overall score. This corresponded with adult learning principles such as addressing multiple learning styles and designing learning objectives to achieve a thorough understanding of concepts and the ability to apply skills in a new context. Training material that incorporated advanced aspects of Bloom’s Taxonomy such as “Apply,” “Analyze,” “Evaluate,” and “Create” also included multiple means of interactivity to foster such learning. Following best practices regarding design and interactivity permitted the top five resources to stand out.

A strength of this research is the thorough search that we conducted for e-learning tools, which we feel confident did not miss relevant resources at the time of the search. However, the challenge of maintaining the currency of our environmental scan in the context of the rapid development of e-learning meant that recently developed or newly published training resources would not be included in this evaluation. Limiting inclusion to resources that encompassed the full process of conducting systematic reviews meant that we did not evaluate educational material teaching one or two steps that can be useful for researchers who need very specific instruction, though some of these resources have been evaluated elsewhere [15].

Another limitation was that our ability to evaluate usability and navigation was limited for a few included resources where we did not have complete access to the entire course or module. Excluding such resources from our scan would have left a gap in the review, but we acknowledge that the evaluation was incomplete and, therefore, the overall score for these resources was biased. Unlike other evaluations of individual e-learning tools concerning systematic reviews, we applied a standardized rubric to all identified resources across multiple domains and, therefore, added to what is known regarding the quality of such resources. Other researchers have evaluated single web-based resources to support systematic review authors but did not compare the training tools against any instructional standards, assess learning outcomes, or compare them to other resources [19, 20].

Although there were no other summative evaluations of research method e-learning tools, our findings of strengths in content and weaknesses in design and interactivity were similar to those of the evaluation of online EBM modules on which we based our work [17]. While Foster et al. also found that resources performed well in the Usability domain [17], our analysis showed only a trend toward strength rather than significance. The outcomes of our evaluation concerning the low use of the levels of Bloom’s Taxonomy [26] and shortcomings in coverage of learning objectives also aligned with an assessment conducted by Boden and Murphy regarding the searching skills instruction in an online systematic review course [16].

Individual online training resources have been evaluated with various methods, including user experience [14], learner performance [15], and ranking of components based on Bloom’s Taxonomy [16]. Of these approaches, objectively assessing outcomes is the most robust means of evaluating instructional effectiveness but would not be feasible to complete for all twenty identified resources. Whereas researchers examining other topics of instruction have compared sets of e-learning resources using an appraisal standard such as the Currency, Reliability, Authority, Accuracy, and Purpose (CRAAP) Test [22], which looks solely at content characteristics, or have simply categorized the learning tools [23], our study applied a detailed rubric across four domains and employed multiple evaluators to increase objectivity while comparing resources to previously identified best practices.

Librarians and other educators who support researchers conducting reviews should keep in mind the strengths and weaknesses of existing systematic review training resources. To be able to recommend appropriate learning tools, they should weigh researchers’ access to funding, time, interest in following a course versus a more self-directed learning format, and point at which the need for instruction arises, in addition to the criteria regarding Content, Design, Interactivity, and Usability applied in our study.

In addition to making recommendations, librarians and other educators should consider these criteria when designing online educational tools about systematic review methods. When developing new e-learning tools, regardless of format, creators should take particular care to include measurable learning objectives and increase interactivity to achieve more levels of Bloom’s Taxonomy [26] to maximize the impact of the training resource. Videos can be useful and are easily accessible learning resources that can be produced with few resources; however, creators should follow better educational practices for e-learning by adding a table of contents or section markers to aid navigation and insert quizzes or other interactive elements to facilitate deeper comprehension. For online courses and modules, navigation bars or a table of contents can help orient users.

Subsequent research would help to illuminate the types of users who would most benefit from different types and formats of instructional material. Other future research includes testing some of the high-quality e-learning resources with independent users and evaluating learner satisfaction and preferences, as well as assessing the rate of completion and quality of the reviews that are produced after the instructional intervention.

This evaluation identifies existing high-quality learning resources from trusted sources that can be recommended to individuals who are seeking to improve their understanding of systematic review methods and increase their skills for conducting evidence syntheses. The courses and web module that had the highest overall evaluation scores included high levels of interactivity and followed good instructional design principles, including measurable learning objectives achieved by a combination of approaches for different learning styles. These resources also used recommended practices regarding usability, such as easy navigation and accessibility for learners with disabilities. Rating the publicly available training tools for systematic review methods using an evaluation framework also encourages reflection on educational material used in systematic review consultations or being developed for instruction. Noting the common limitations of online courses, web modules, and video tutorials will inform efforts to develop new resources by keeping in mind important elements of content, design, interactivity, and usability.

## SUPPLEMENTAL FILES

Appendix A Environmental scan searches

Appendix B Scoring rubric

Appendix C Full list of evaluated resources, alphabetically by host

Appendix D Exact evaluation scores for each resource
