# Supplementary material for: Environmental scan and evaluation of best practices for online systematic review resources
Source: J Med Libr Assoc. 2018 Apr 1;106(2):208–18. doi: 10.5195/jmla.2018.241 (PMC5886503; doi:10.5195/jmla.2018.241)
Supplement: Appendix A [file jmla-106-208-s001.pdf]

JMLA Journal of the Medical Library Association

SC

Supplemental content to
J Med Libr Assoc. Apr;106(2):dx.doi.org/10.5195/jmla.2018.241
www.jmla.mlanet.org
© Parker, Boulos, Visintini, Ritchie, Hayden 2018

Environmental scan and evaluation of best practices for online systematic review resources

Robin M. N. Parker, MLIS; Leah Boulos; Sarah Visintini; Krista Ritchie; Jill Hayden

APPENDIX A

Environmental scan searches

Websites searched

("systematic review" OR "comprehensive review" OR "scoping review" OR "evidence review" OR "knowledge synthesis" OR "evidence synthesis") AND (train OR training OR education OR instruction OR learn OR learning OR course OR workshop OR seminar OR class site):[INSERT SITE]

Knowledge synthesis organizations	Website
Agency for Healthcare Research and Quality (AHRQ)	www.ahrq.gov
Alberta College of Family Physicians (ACFP)	https://www.acfp.ca/tools-for-practice/
Alberta Health: Health Technology Assessment Provincial Reviews—Ongoing and Complete	www.health.alberta.ca/initiatives/AHTDP-reviews.html
Australian Government Department of Health and Ageing: Australia and New Zealand Horizon Scanning Network (ANZHSN)	www.horizonscanning.gov.au/internet/horizon/publishing.nsf/Content/technologies-assessed-lp-2
Australian Government Department of Health and Ageing: Medical Services Advisory Committee (MSAC)	www.msac.gov.au/internet/msac/publishing.nsf/Content/completed-assessments
Australian Government Department of Health and Ageing: Pharmaceuticals Benefits Scheme	www.pbs.gov.au/browse/medicine-listing
Best Evidence Medical Education (BEME) Collaboration	www.bemecollaboration.org
Blue Cross and Blue Shield Association Technology Evaluation Center (TEC)	https://www.bcbs.com/node/1516
California Technology Assessment Forum (CTAF)	ctaf.org
Campbell Collaboration	www.campbellcollaboration.org
Canadian Agency for Drugs and Technologies in Health (CADTH)	https://www.cadth.ca
Centers for Medicare & Medicaid Services Technology Assessments	www.cms.gov/medicare-coverage-database/indexes/technology-assessments-index.aspx?TAId=85&bc=AAAQAAAAAAAA&
EuroScan International Network	www.euroscan.org.uk
Evidence for Policy and Practice Information and Co-ordinating Centre (EPPI-Centre)	eppi.ioe.ac.uk/cms/

JMLA Journal of the Medical Library Association

SC

Supplemental content to
J Med Libr Assoc. Apr;106(2):dx.doi.org/10.5195/jmla.2018.241
www.jmla.mlanet.org
© Parker, Boulos, Visintini, Ritchie, Hayden 2018

Knowledge synthesis organizations	Website
Federal Reserve Bank of St. Louis: Economic Research Division: IDEAS Database	ideas.repec.org
Health Information and Quality Authority (Ireland): Health Technology Assessment	www.hiqa.ie/healthcare/health-technology-assessment/assessments
Health Quality Council of Alberta (HQCA)	www.hqca.ca
Health Quality Ontario	www.hqontario.ca/evidence/publications-and-ohtac-recommendations
Health Services Executive: Irish Health Repository (Lenus)	www.lenus.ie/hse/
Healthcare Improvement Scotland	www.healthcareimprovementscotland.org
INAHTA	www.inahta.org
Institut national d'excellence en santé et en services sociaux	www.inesss.qc.ca/index.php?id=49&L=1
Institute for Clinical and Economic Review (ICER)	www.icer-review.org/
Institute of Health Economics (IHE)	www.ihe.ca
International Society for Pharmacoeconomics and Outcomes Research (ISPOR): *Value in Health*	www.valueinhealthjournal.com/issues
Joanna Briggs Institute	joannabriggs.org
Knowledge Translation Canada (KT Canada)	ktclearinghouse.ca/ktcanada
Manitoba Centre for Health Policy: Deliverables	mchp-appserv.cpe.umanitoba.ca/deliverablesList.html
McGill University Health Centre (MUHC): Technology Assessment Unit of the MUHC	www.mcgill.ca/tau/publications/
McMaster University: Centre for Health Economics and Policy Analysis (CHEPA)	www.chepa.org/research-papers/faculty-publications
Monash Health Centre for Clinical Effectiveness (CCE)	www.monashhealth.org/page/CCE
National Centre for Pharmacoeconomics: Pharmacoeconomic Evaluations	www.ncpe.ie/pharmacoeconomic-evaluations/
National Collaborating Centre for Methods and Tools (NCCMT)	www.nccmt.ca
National Institute for Health and Care Excellence (NICE)	www.nice.org.uk
National Institute for Health Research (NIHR): Evaluation, Trials and Studies Coordinating Centre (NETSCC)	www.southampton.ac.uk/netscc/

JMLA Journal of the Medical Library Association

SC
Supplemental content to
J Med Libr Assoc. Apr;106(2):dx.doi.org/10.5195/jmla.2018.241
www.jmla.mlanet.org
© Parker, Boulos, Visintini, Ritchie, Hayden 2018

Knowledge synthesis organizations	Website
National Institute for Health Research (NIHR): Horizon Scanning Research & Intelligence Centre (NHSC)	www.invo.org.uk/posttypelinks/national-institute-for-health-research-national-horizon-scanning-centre/
National Prescribing Service (NPS RADAR)	www.nps.org.au/publications/health-professional/nps-radar
NHS Purchasing and Supply Agency Centre for Evidence-based Purchasing (CEP)	nhscep.useconnect.co.uk/CEPProducts/Catalogue.aspx
Newfoundland and Labrador Centre for Applied Health Research (NLCAHR)	www.nlcahr.mun.ca/CHRSP/CompletedCHRSP.php
Ottawa Hospital Research Institute (OHRI): Knowledge Synthesis Group	www.ohri.ca/ksgroup/publications.asp
Ontario Ministry of Health and Long-Term Care: Ontario Case Costing Initiative (OCCI)	www.health.gov.on.ca/en/
Program for Assessment of Technology in Health (PATH)	https://www.path-hta.ca/research
Public Health Agency of Canada: Economic Burden of Illness in Canada	www.phac-aspc.gc.ca/ebic-femc/index-eng.php
Public Health Wales	www.attract.wales.nhs.uk
Queensland Government Health Policy Advisory Committee on Technology (HealthPACT)	https://www.health.qld.gov.au
Royal Australasian College of Surgeons: Australian Safety and Efficacy Register of New Interventional Procedures - Surgical (ASERNIP)	www.surgeons.org/for-health-professionals/audits-and-surgical-research/asernip-s/
Sick Kids Paediatric Economic Database Evaluation (PEDE)	pede.ccb.sickkids.ca/pede/search.jsp
Therapeutics Initiative: Therapeutics Letter	www.ti.ubc.ca/TherapeuticsLetter
Toronto Health Economics and Technology Assessment Collaborative (THETA)	theta.utoronto.ca/content.php?pid=411861&sid=3372336
University of Aberdeen Health Economics Research Unit (HERU)	https://www.abdn.ac.uk/heru/
University of British Columbia Centre for Health Services and Policy Research	chspr.ubc.ca
University of York: Centre for Reviews and Dissemination (CRD)	https://www.york.ac.uk/crd/
Washington State Health Care Authority	https://www.hca.wa.gov
World Health Organization Regional Office for Europe: Health Evidence Network (WHO HEN)	www.euro.who.int/en/data-and-evidence/evidence-informed-policy-making/health-evidence-network-hen
