# Supplementary material for: Environmental scan and evaluation of best practices for online systematic review resources
Source: J Med Libr Assoc. 2018 Apr 1;106(2):208–18. doi: 10.5195/jmla.2018.241 (PMC5886503; doi:10.5195/jmla.2018.241)
Supplement: Appendix B [file jmla-106-208-s002.pdf]

JMLA Journal of the Medical Library Association

Supplemental content to
J Med Libr Assoc. Apr;106(2):dx.doi.org/10.5195/jmla.2018.241
www.jmla.mlanet.org
© Parker, Boulos, Visintini, Ritchie, Hayden 2018

Environmental scan and evaluation of best practices for online systematic review resources

Robin M. N. Parker, MLIS; Leah Boulos; Sarah Visintini; Krista Ritchie; Jill Hayden

APPENDIX B

Scoring rubric

Domain	Question	Possible answers	Points awarded
Content	1. What topics are covered by the module?	Defining a research question/developing a protocol	0
		Rigorous search process	0
		Selection criteria	0
		Critical appraisal or risk of bias assessment	0
		Data extraction	0
		Analysis/presenting findings	0
		Can't tell from available info	0
Content	2. A) Is the content credible? ("The information should be trustworthy and convincing. This means the provided information should be based on reliable sources and fitted with complete references." QuADEM Manual p. 38)	No (organization not known for work in SR and no citations to back up content)	0
		Somewhat (credible provider, but no citations within resource)	0.5
		Yes (credible provider and provides citations for content)	1
		Can't tell from available info	0
Content	2. B) Is the content relevant?	No (content does not align with purpose, learning objectives, or audience)	0
		Somewhat (content aligns somewhat with purpose, learning objectives, and audience)	0.5
		Yes (content is adapted to purpose, learning objectives, and audience)	1
		Can't tell from available info	0

JMLA Journal of the Medical Library Association

SC
Supplemental content to
J Med Libr Assoc. Apr;106(2):dx.doi.org/10.5195/jmla.2018.241
www.jmla.mlanet.org
© Parker, Boulos, Visintini, Ritchie, Hayden 2018

Domain	Question	Possible answers	Points awarded
Content	2. C) Is the content current? ("It should be clearly stated when the web content was produced, when it was updated, and whether it is still valid. A regular update of links is also a must." QuADEM Manual p. 38)	No (no indication of creation or update date, site is not modern looking, e.g. think web 1.0 vs. web 2.0)<br>Somewhat (if an indication of date is given, is between 5 and 10 years old, or if no date is given but site looks modern)<br>Yes (is less than 5 years old, date is provided or can be inferred)<br>Can't tell from available info	0<br>0.5<br>1<br>0
Content	2. D) Is the content well organized? ("The information provided by a digital learning module should be logically organized and well-structured. The way the information is organized and structured should be clearly visible to the module's users, with titles, subtitles, paragraphs, links between text parts, and connections to other relevant sections within the module." QuADEM Manual p. 38)	No (disorganized)<br>Somewhat (neither disorganized nor very organized)<br>Yes (very organized)<br>Can't tell from available info	0<br>0.5<br>1<br>0
Content	2. E) Is the content easily understood? ("The target audience should be able to understand the [theoretical] information provided. This means the prior knowledge of the target audience needs to be taken into account when compiling the content of the learning module." QuADEM Manual p. 38)	No<br>Somewhat<br>Yes<br>Can't tell from available info	0<br>0.5<br>1<br>0
Content	2. F) Is the content focused and specific? ("The information offered should be detailed and focused. This can be promoted by including a summary of the highlights of the theoretical knowledge necessary to achieve the learning objectives. While the given information should address a well specified subject, it should also include the background knowledge for contextualisation." QuADEM Manual p. 38)	No<br>Somewhat<br>Yes<br>Can't tell from available info	0<br>0.5<br>1<br>0

JMLA Journal of the Medical Library Association

SC

Supplemental content to
J Med Libr Assoc. Apr;106(2):dx.doi.org/10.5195/jmla.2018.241
www.jmla.mlanet.org
© Parker, Boulos, Visintini, Ritchie, Hayden 2018

Domain	Question	Possible answers	Points awarded
Content	2. G) Is the content appropriate style/language for audience? ("It is important that the formulation and presentation of the information fits in with the prior knowledge of the audience. Academic/professional jargon that might not be familiar to the target audience should be avoided. Different audiences need to be addressed differently. For example, one could apply a more formal style when addressing teachers and a more informal style when addressing students. However, it is always best to address the audience directly and use active formulations." QuADEM Manual p. 42)	No (language is not appropriate for audience, e.g., very technical as introductory resource with no supporting definitions)<br>Somewhat (language is probably appropriate even if no audience is provided, neither too simplistic nor too much jargon)<br>Yes (language is appropriate to audience)<br>Can't tell from available info	0<br>0.5<br>1<br>0
Design	3. What levels of Bloom's Taxonomy are covered? Select all that apply. See Bloom's Revised Taxonomy (2001): https://cft.vanderbilt.edu/guides-sub-pages/blooms-taxonomy/	Remember (recall facts and basic concepts)<br>Understand (explain ideas or concepts)<br>Apply (use information in new situations)<br>Analyze (draw connections among ideas)<br>Evaluate (justify a stand or decision)<br>Create (produce new or original work)<br>Can't tell from available info	1<br>1<br>1<br>1<br>1<br>1<br>0
Design	4. A) Does the module explain its purpose and audience?	No (no explanation given)<br>Somewhat (some explanation provided, might be vague)<br>Yes (purpose and audience specifically identified)<br>Can't tell from available info	0<br>0.5<br>1<br>0
Design	4. B) Does the module provide measurable objectives?	No (no learning objectives provided)<br>Somewhat (learning objectives are vague and difficult to quantify)<br>Yes (learning objectives are specific, e.g., describe a specific task that users will be able to complete)<br>Can't tell from available info	0<br>0.5<br>1<br>0

Supplemental content to
J Med Libr Assoc. Apr;106(2):dx.doi.org/10.5195/jmla.2018.241
www.jmla.mlanet.org
© Parker, Boulos, Visintini, Ritchie, Hayden 2018

Domain	Question	Possible answers	Points awarded
Design	4. C) Does the module cover all objectives?	No (no learning objectives provided, or module does not align with learning objectives)	0
		Somewhat (content may be able to support learning objectives, but may be dubious, e.g., a 1-hour module that states learners will be able to perform review afterwards)	0.5
		Yes (content sufficient to cover learning objectives)	1
		Can't tell from available info	0
Design	5. What learning styles are incorporated? Select all that apply.	Visual (e.g., videos, graphics, charts)	1
		Audio (e.g., recordings or video with sound)	1
		Spatial (e.g., puzzles, drag and drop, matching up items)	1
		Can't tell from available info	0
Interactivity	6. What is the level of interactivity?	None (e.g., a video)	0
		Some (e.g., clicking to get to the next module)	0.5
		High (e.g., incorporation of several activities: quizzes, clicking, scrolling, etc.)	1
		Can't tell from available info	0
Interactivity	7. What types of interaction are there? Select all that apply.	None	0
		Clicking	1
		Performing search	1
		Scrolling	1
		Taking a survey/quiz	1
		Type in answers or post to discussion board	1
		Other:	1
		Can't tell from available info	0

Supplemental content to
J Med Libr Assoc. Apr;106(2):dx.doi.org/10.5195/jmla.2018.241
www.jmla.mlanet.org
© Parker, Boulos, Visintini, Ritchie, Hayden 2018

Domain	Question	Possible answers	Points awarded
Interactivity	8. A) Is the interactivity relevant?	No (answer if there is no interactivity)	0
		Somewhat (clicking or scrolling at a minimum)	0.5
		Yes (interactivity furthers learning objectives, e.g., makes you test your knowledge)	1
		Can't tell from available info	0
Interactivity	8. B) Does the interactivity have an appropriate level of difficulty?	No (answer if there is no interactivity or inappropriate level of difficulty: too easy or too hard)	0
		Somewhat (interactivity that keeps you awake)	0.5
		Yes (interactivity promotes learning)	1
		Can't tell from available info	0
Interactivity	8. C) Does the interactivity provide feedback?	No (answer if there is no interactivity, or if there is no feedback)	0
		Somewhat (this is an unlikely answer, try to answer Yes or No)	0.5
		Yes (does provide feedback, e.g., answers to a quiz or exercise, response from facilitator or peers)	1
		Can't tell from available info	0
Interactivity	8. D) Does the interactivity provide a place to reflect on learning?	No (answer if there is no interactivity or no place to reflect on learning)	0
		Somewhat (this is an unlikely answer, try to answer Yes or No)	0.5
		Yes (e.g., discussion boards, forums or assignments)	1
		Can't tell from available info	0
Usability	9. Is the layout appealing? ("If users consider the module not visually appealing it will negatively influence their perception of the module. To make the module more appealing: Use a limited number of colours; Use bright	No (extremely busy or extremely boring)	0
		Somewhat	0.5
		Yes (streamlined look, text broken up through formatting or with use of images)	1

Journal of the Medical Library Association
Supplemental content to
J Med Libr Assoc. Apr;106(2):dx.doi.org/10.5195/jmla.2018.241
www.jmla.mlanet.org
© Parker, Boulos, Visintini, Ritchie, Hayden 2018

Domain	Question	Possible answers	Points awarded
	colours sparsely; Do not overuse pictures; Do not avert attention from the main content; The idea of what is appealing is subjective and is ideally verified in a focus group." QuADEM Manual p. 52)	Can't tell from available info	0
Usability	10. Is it easy to navigate?	No (if we can get lost navigating it, then others most certainly will)	0
		Somewhat (may not have breadcrumbs, but still fairly intuitive, unless you want to jump back to beginning or navigate in a way it likely wasn't intended)	0.5
		Yes (use of breadcrumbs, navigation pane or other indicators, or can jump all over the place without getting lost)	1
		Can't tell from available info	0
Usability	11. Do learners determine their own learning path?	No (no control over content; this answer is probably rare)	0
		Somewhat (e.g., long video that isn't divided into sections; user can try to scroll through video but difficult to find content they want)	0.5
		Yes (content is organized in such a way that the user can click or jump ahead to content that is most relevant to them or pause it)	1
		Can't tell from available info	0
Usability	12. A) Does it meet the requirement of the Americans with Disabilities Act with low use of red/green colours?	Yes (no red/green, or use of red/green where won't affect reading or understanding of content)	1
		No (there is a lot of red/green, or use of red/green where it might confuse the reader, such as green text on red)	0
		Does not apply	1
		Can't tell from available info	0

SC
Supplemental content to
J Med Libr Assoc. Apr;106(2):dx.doi.org/10.5195/jmla.2018.241
www.jmla.mlanet.org
© Parker, Boulos, Visintini, Ritchie, Hayden 2018

Domain	Question	Possible answers	Points awarded
Usability	12. B) Does it meet the requirement of the Americans with Disabilities Act by including captions?	Yes (use of captions in video, or sufficiently detailed slides going along with audio, or availability of transcript)	1
		No (no captions, minimal slides, no transcript; in other words, easy to get lost if audio unclear)	0
		Does not apply (non-audio-based content, e.g., text-based web module)	1
		Can't tell from available info	0
Usability	12. C) Does it meet the requirement of the Americans with Disabilities Act with consistent navigation?	Yes (e.g., use of breadcrumbs, navigation pane, or simple "Next Page" features)	1
		No (circuitous or unclear navigation)	0
		Does not apply (e.g., for videos, no navigation required)	1
		Can't tell from available info	0
Usability	12. D) Does it meet the requirement of the Americans with Disabilities Act with audio that turns off?	Yes	1
		No	0
		Does not apply (not audio-based resource)	1
		Can't tell from available info	0
Usability	12. E) Does it meet the requirement of the Americans with Disabilities Act with option for user to pause?	Yes	1
		No	0
		Does not apply (not audio- or video-based resource)	1
		Can't tell from available info	0
Overall evaluation notes	13. Provide any additional notes about the resource/your evaluation here.		0
