# Supplementary material for: Environmental scan and evaluation of best practices for online systematic review resources
Source: J Med Libr Assoc. 2018 Apr 1;106(2):208–18. doi: 10.5195/jmla.2018.241 (PMC5886503; doi:10.5195/jmla.2018.241)
Supplement: Appendix C [file jmla-106-208-s003.pdf]

JMLA Journal of the Medical Library Association

SC

Supplemental content to
J Med Libr Assoc. Apr;106(2):dx.doi.org/10.5195/jmla.2018.241
www.jmla.mlanet.org
© Parker, Boulos, Visintini, Ritchie, Hayden 2018

Environmental scan and evaluation of best practices for online systematic review resources

Robin M. N. Parker, MLIS; Leah Boulos; Sarah Visintini; Krista Ritchie; Jill Hayden

APPENDIX C

Full list of evaluated resources, alphabetically by host

Host	Name of resource
Online courses	
Coursera/Johns Hopkins Bloomberg School of Public Health	Introduction to Systematic Review and Meta-Analysis
Dalla Lana School of Public Health, University of Toronto/Knowledge Translation Program, St. Michael's Hospital	Introduction to Systematic Review and Meta-Analysis Course
EPPI-Centre	Systematic Reviews: Meta-Analysis, Qualitative Synthesis, and Mixed Mode Synthesis*
EPPI-Centre	Systematic Reviews: Diversity, Design and Debate†
Joanna Briggs Institute	Comprehensive Systematic Review Training Program (CSRTP)*
National Center for the Dissemination of Disability Research	Developing Evidence-Based Products Using the Systematic Review Process
University College London (UCL) Life Learning	Systematic Reviews 1: Introduction<br>Systematic Reviews 2: Analysing Data<br>Systematic Reviews 3: Interpreting Data†
Web modules	
Cochrane Training	Online Learning Modules for Cochrane Authors*
Evidence-Based Behavioral Practice	Introduction to Systematic Reviews
National Certification Corporation	WB1619 Systematic Review to Support Evidence Based Practice
Teach Epi: A Website for Teaching and Learning Epidemiology	EPIB-672: Systematic Reviews and Meta-Analyses
University of Edinburgh Centre for Cognitive Ageing and Cognitive Epidemiology	Systematic Reviews and Meta-Analyses: A Step-by-Step Guide
Videos	
Campbell Collaboration	Campbell Collaboration Training Videos
Center on Knowledge Translation for Disability and Rehabilitation Research (KTDRR)	Qualitative Research Synthesis: KTDRR's Web-based Workshop Series
Complete Sports Care/Queen Mary University of London	Introduction to Completing A Systematic Review
Knowledge Translation for Employment Research Center	Knowledge Production Methods†
methods@manchester: University of Manchester	What Is Systematic Reviews by Helen Worthington
Neurosurgery Research & Education Foundation	Systematic Reviews and Meta-Analysis

Journal of the Medical Library Association

Supplemental content to
J Med Libr Assoc. Apr;106(2):dx.doi.org/10.5195/jmla.2018.241
www.jmla.mlanet.org
© Parker, Boulos, Visintini, Ritchie, Hayden 2018

Host	Name of resource
Research Centers in Minority Institutions (RCMI) Program University of Puerto Rico (UPR) Medical Sciences Campus	Systematic Review and Meta-Analysis Workshop (two parts)
Syrian American Medical Society (SAMS) Education Committee	SAMS Evidence-Based Medicine (EBM) Course: Systematic Reviews 1 & 2, Hassan Murad (two parts)

* Name of resource has changed since evaluation was completed.

† No longer available as evaluated.
