# Supplementary material for: Environmental scan and evaluation of best practices for online systematic review resources
Source: J Med Libr Assoc. 2018 Apr 1;106(2):208–18. doi: 10.5195/jmla.2018.241 (PMC5886503; doi:10.5195/jmla.2018.241)
Supplement: Appendix D [file jmla-106-208-s004.pdf]

JMLA Journal of the Medical Library Association

Supplemental content to
J Med Libr Assoc. Apr;106(2):dx.doi.org/10.5195/jmla.2018.241
www.jmla.mlanet.org
© Parker, Boulos, Visintini, Ritchie, Hayden 2018

Environmental scan and evaluation of best practices for online systematic review resources

Robin M. N. Parker, MLIS; Leah Boulos; Sarah Visintini; Krista Ritchie; Jill Hayden

APPENDIX D

Exact evaluation scores for each resource

	Content								Design					Interactivity						Usability								Total score	
**Resource #**	**1.**	**2. A)**	**2. B)**	**2. C)**	**2. D)**	**2. E)**	**2. F)**	**2. G)**	**3.**	**4. A)**	**4. B)**	**4. C)**	**5.**	**6.**	**7.**	**8. A)**	**8. B)**	**8. C)**	**8. D)**	**9.**	**10.**	**11.**	**12. A)**	**12. B)**	**12. C)**	**12. D)**	**12. E)**	**n/37**	**(%)**
R01	Defining a research question/Developing a protocol<br>Rigorous search process<br>Selection criteria<br>Critical appraisal or risk of bias assessment<br>Data extraction<br>Presenting findings/analysis	1	1	1	1	1	1	1	4	1	1	1	2	1	5	1	1	1	1	1	1	1	1	1	1	1	1	34/37	(92%)
R02	Defining a research question/Developing a protocol<br>Rigorous search process<br>Selection criteria<br>Critical appraisal or risk of bias assessment<br>Data extraction<br>Presenting findings/analysis	1	1	1	1	1	1	0.5	5	0.5	1	1	2	1	4	1	1	1	1	1	1	1	1	1	1	1	1	33/37	(89%)

JMLA Journal of the Medical Library Association

Supplemental content to
J Med Libr Assoc. Apr;106(2):dx.doi.org/10.5195/jmla.2018.241
www.jmla.mlanet.org
© Parker, Boulos, Visintini, Ritchie, Hayden 2018

**Resource #**	**Content**									**Design**				**Interactivity**						**Usability**								**Total score**	
	**1.**	**2. A)**	**2. B)**	**2. C)**	**2. D)**	**2. E)**	**2. F)**	**2. G)**	**3.**	**4. A)**	**4. B)**	**4. C)**	**5.**	**6.**	**7.**	**8. A)**	**8. B)**	**8. C)**	**8. D)**	**9.**	**10.**	**11.**	**12. A)**	**12. B)**	**12. C)**	**12. D)**	**12. E)**	**n/37**	**(%)**
R03	Defining a research question/Developing a protocol<br>Rigorous search process<br>Critical appraisal or risk of bias assessment<br>Data extraction<br>Presenting findings/analysis	1	1	0.5	1	1	1	1	6	1	1	1	2	1	3	1	1	1	0	1	1	1	1	1	1	1	1	32.5/37	(88%)
R04	Defining a research question/Developing a protocol<br>Rigorous search process<br>Selection criteria<br>Critical appraisal or risk of bias assessment<br>Data extraction<br>Presenting findings/analysis	1	1	1	1	1	1	0.5	6	0.5	1	1	2	1	5	1	1	1	1	1	0	0.5	1	0	0	0	0	29.5/37	(80%)

Supplemental content to
J Med Libr Assoc. Apr;106(2):dx.doi.org/10.5195/jmla.2018.241
www.jmla.mlanet.org
© Parker, Boulos, Visintini, Ritchie, Hayden 2018

Resource #	Content								Design					Interactivity						Usability								Total score	
	**1.**	**2. A)**	**2. B)**	**2. C)**	**2. D)**	**2. E)**	**2. F)**	**2. G)**	**3.**	**4. A)**	**4. B)**	**4. C)**	**5.**	**6.**	**7.**	**8. A)**	**8. B)**	**8. C)**	**8. D)**	**9.**	**10.**	**11.**	**12. A)**	**12. B)**	**12. C)**	**12. D)**	**12. E)**	**n/37**	**(%)**
R05	Defining a research question/Developing a protocol<br>Rigorous search process<br>Selection criteria<br>Critical appraisal or risk of bias assessment<br>Data extraction<br>Presenting findings/analysis	1	1	1	1	1	0	1	6	0.5	1	1	2	1	5	1	1	1	1	0	0	1	0	0	0	0	0	27.5/37	(74%)
R06	Defining a research question/Developing a protocol<br>Rigorous search process<br>Selection criteria<br>Data extraction<br>Presenting findings/analysis	1	1	1	1	0	0	0	3	1	1	0	2	1	4	1	1	1	1	1	1	1	0	0	1	1	1	26/37	(70%)

JMLA Journal of the Medical Library Association

Supplemental content to
J Med Libr Assoc. Apr;106(2):dx.doi.org/10.5195/jmla.2018.241
www.jmla.mlanet.org
© Parker, Boulos, Visintini, Ritchie, Hayden 2018

Resource #	Content								Design					Interactivity						Usability								Total score	
	1.	2. A)	2. B)	2. C)	2. D)	2. E)	2. F)	2. G)	3.	4. A)	4. B)	4. C)	5.	6.	7.	8. A)	8. B)	8. C)	8. D)	9.	10.	11.	12. A)	12. B)	12. C)	12. D)	12. E)	n/37	(%)
R07	Defining a research question/Developing a protocol<br>Rigorous search process<br>Selection criteria<br>Critical appraisal or risk of bias assessment<br>Data extraction<br>Presenting findings/analysis	1	1	0.5	1	1	1	1	3	1	0	0	2	1	2	1	1	1	0	1	1	1	1	1	1	0	0	24.5/37	(66%)
R08	Defining a research question/Developing a protocol<br>Rigorous search process<br>Selection criteria<br>Critical appraisal or risk of bias assessment<br>Data extraction<br>Presenting findings/analysis	1	1	1	0.5	1	1	1	2	1	0	0	2	0.5	2	0.5	0	0	0	0.5	0.5	0.5	1	1	1	1	1	21/37	(57%)

JMLA Journal of the Medical Library Association

SC
Supplemental content to
J Med Libr Assoc. Apr;106(2):dx.doi.org/10.5195/jmla.2018.241
www.jmla.mlanet.org
© Parker, Boulos, Visintini, Ritchie, Hayden 2018

	**Content**								**Design**					**Interactivity**						**Usability**								**Total score**	
**Resource #**	**1.**	**2. A)**	**2. B)**	**2. C)**	**2. D)**	**2. E)**	**2. F)**	**2. G)**	**3.**	**4. A)**	**4. B)**	**4. C)**	**5.**	**6.**	**7.**	**8. A)**	**8. B)**	**8. C)**	**8. D)**	**9.**	**10.**	**11.**	**12. A)**	**12. B)**	**12. C)**	**12. D)**	**12. E)**	**n/37**	**(%)**
R09	Defining a research question/Developing a protocol<br>Rigorous search process<br>Selection criteria<br>Critical appraisal or risk of bias assessment<br>Data extraction<br>Presenting findings/analysis	1	1	1	0.5	1	1	1	1	1	0.5	0.5	2	0.5	2	0.5	0.5	0	0	0.5	0	0.5	1	1	1	1	1	21/37	(57%)
R10	Defining a research question/Developing a protocol<br>Rigorous search process<br>Selection criteria<br>Critical appraisal or risk of bias assessment<br>Data extraction<br>Presenting findings/analysis	1	1	1	0	0	0.5	1	2	1	1	0.5	1	0.5	3	1	1	1	0	0.5	0	1	0	1	0	1	1	21/37	(57%)
R11	Rigorous search process<br>Critical appraisal or risk of bias assessment<br>Presenting findings/analysis	0.5	1	1	1	0.5	1	0.5	2	1	1	1	2	0	1	0.5	0.5	0.5	0	1	0.5	0.5	1	0	1	1	1	21/37	(57%)

JMLA Journal of the Medical Library Association

Supplemental content to
J Med Libr Assoc. Apr;106(2):dx.doi.org/10.5195/jmla.2018.241
www.jmla.mlanet.org
© Parker, Boulos, Visintini, Ritchie, Hayden 2018

Resource #	Content								Design					Interactivity						Usability								Total score	
	**1.**	**2. A)**	**2. B)**	**2. C)**	**2. D)**	**2. E)**	**2. F)**	**2. G)**	**3.**	**4. A)**	**4. B)**	**4. C)**	**5.**	**6.**	**7.**	**8. A)**	**8. B)**	**8. C)**	**8. D)**	**9.**	**10.**	**11.**	**12. A)**	**12. B)**	**12. C)**	**12. D)**	**12. E)**	**n/37**	**(%)**
R12	Defining a research question/Developing a protocol<br>Rigorous search process<br>Selection criteria<br>Critical appraisal or risk of bias assessment<br>Data extraction<br>Presenting findings/analysis	1	1	1	1	1	1	1	1	0.5	0	0	2	0.5	2	0.5	0.5	0	0	0.5	0.5	0.5	1	1	1	1	1	20.5/37	(55%)
R13	Critical appraisal or risk of bias assessment<br>Data extraction<br>Presenting findings/analysis	1	0	1	1	0	0	0	6	1	1	0	0	1	4	1	1	1	1	0	0	0	0	0	0	0	0	20/37	(54%)
R14	Defining a research question/Developing a protocol<br>Rigorous search process<br>Critical appraisal or risk of bias assessment<br>Data extraction<br>Presenting findings/analysis	1	1	1	1	0.5	1	0.5	1	0.5	0	0	1	0.5	2	0.5	0.5	0	0	1	1	1	1	1	1	1	1	20/37	(54%)

JMLA Journal of the Medical Library Association

Supplemental content to
J Med Libr Assoc. Apr;106(2):dx.doi.org/10.5195/jmla.2018.241
www.jmla.mlanet.org
© Parker, Boulos, Visintini, Ritchie, Hayden 2018

Resource #	Content								Design				Interactivity						Usability								Total score	
	**1.**	**2. A)**	**2. B)**	**2. C)**	**2. D)**	**2. E)**	**2. F)**	**2. G)**	**3.**	**4. A)**	**4. B)**	**4. C)**	**5.**	**6.**	**7.**	**8. A)**	**8. B)**	**8. C)**	**8. D)**	**9.**	**10.**	**11.**	**12. A)**	**12. B)**	**12. C)**	**12. D)**	**12. E)**	**n/37**	**(%)**
R15	Defining a research question/Developing a protocol<br>Rigorous search process<br>Selection criteria<br>Critical appraisal or risk of bias assessment<br>Data extraction<br>Presenting findings/analysis	1	1	1	1	1	0.5	0.5	1	0.5	0	0	2	0.5	2	0	0	0	0	0.5	1	1	1	1	1	1	1	19.5/37	(53%)
R16	Defining a research question/Developing a protocol<br>Rigorous search process<br>Selection criteria<br>Critical appraisal or risk of bias assessment<br>Data extraction<br>Presenting findings/analysis	1	1	0.5	0.5	1	1	1	1	1	1	1	2	0.5	2	0	0	0	0	0	0.5	1	0	0	1	1	1	19/37	(51%)

JMLA Journal of the Medical Library Association

SC
Supplemental content to
J Med Libr Assoc. Apr;106(2):dx.doi.org/10.5195/jmla.2018.241
www.jmla.mlanet.org
© Parker, Boulos, Visintini, Ritchie, Hayden 2018

Resource #	Content								Design					Interactivity						Usability								Total score	
	**1.**	**2. A)**	**2. B)**	**2. C)**	**2. D)**	**2. E)**	**2. F)**	**2. G)**	**3.**	**4. A)**	**4. B)**	**4. C)**	**5.**	**6.**	**7.**	**8. A)**	**8. B)**	**8. C)**	**8. D)**	**9.**	**10.**	**11.**	**12. A)**	**12. B)**	**12. C)**	**12. D)**	**12. E)**	**n/37**	**(%)**
R17	Defining a research question/Developing a protocol<br>Rigorous search process<br>Selection criteria<br>Critical appraisal or risk of bias assessment<br>Presenting findings/analysis	0.5	1	1	0.5	1	1	1	1	1	0.5	0.5	2	0	0	0	0	0	0	0.5	0.5	0.5	1	0	1	1	1	16.5/37	(45%)
R18	Defining a research question/Developing a protocol<br>Rigorous search process<br>Selection criteria	0.5	1	1	1	1	1	1	1	0.5	0	0	2	0	0	0	0	0	0	1	0.5	0.5	1	0	1	1	1	16/37	(43%)
R19	Defining a research question/Developing a protocol<br>Rigorous search process<br>Selection criteria<br>Critical appraisal or risk of bias assessment<br>Data extraction<br>Presenting findings/analysis	1	0.5	1	0.5	1	0.5	0.5	1	0	0	0	2	0	0	0	0	0	0	0.5	0.5	0.5	1	0	1	1	1	13.5/37	(36%)

JMLA Journal of the Medical Library Association

Supplemental content to
J Med Libr Assoc. Apr;106(2):dx.doi.org/10.5195/jmla.2018.241
www.jmla.mlanet.org
© Parker, Boulos, Visintini, Ritchie, Hayden 2018

Resource #	Content								Design					Interactivity						Usability								Total score	
	**1.**	**2. A)**	**2. B)**	**2. C)**	**2. D)**	**2. E)**	**2. F)**	**2. G)**	**3.**	**4. A)**	**4. B)**	**4. C)**	**5.**	**6.**	**7.**	**8. A)**	**8. B)**	**8. C)**	**8. D)**	**9.**	**10.**	**11.**	**12. A)**	**12. B)**	**12. C)**	**12. D)**	**12. E)**	**n/37**	**(%)**
R20	Defining a research question/Developing a protocol<br>Selection criteria<br>Critical appraisal or risk of bias assessment<br>Data extraction<br>Presenting findings/analysis	1	0.5	1	0	0.5	0.5	0.5	1	0	0	0	2	0	0	0	0	0	0	0.5	0.5	0.5	1	0	1	1	1	12.5/37	(34%)
